# Real-Life Data on the Efficacy of Canakinumab in Patients with Adult-Onset Still's Disease

**DOI:** 10.1155/2020/8054961

**Published:** 2020-10-15

**Authors:** Antonio Vitale, Virginia Berlengiero, Jurgen Sota, Luisa Ciarcia, Nicola Ricco, Sara Barneschi, Mariam Mourabi, Giuseppe Lopalco, Chiara Marzo, Francesca Bellisai, Florenzo Iannone, Bruno Frediani, Luca Cantarini

**Affiliations:** ^1^Research Center of Systemic Autoinflammatory Diseases, Behçet's Disease and Rheumatology-Ophthalmology Collaborative Uveitis Center, Rheumatology Unit, Department of Medical Sciences, Surgery and Neurosciences, University of Siena, Siena, Italy; ^2^Rheumatology Unit, Department of Emergency and Organ Transplantation, University of Bari, Bari, Italy

## Abstract

**Background:**

Interleukin-1 inhibition has revealed to be a successful treatment approach for patients with adult-onset Still's disease (AOSD). However, real-life experience is focused on the use of anakinra, while data about canakinumab (CAN) are mainly based on case reports and small case series. *Patients and Methods*. Patients classified with AOSD according to Yamaguchi criteria and treated with CAN were consecutively enrolled. Their clinical and therapeutic data were retrospectively collected and statistically analysed to assess the role of CAN as a therapeutic opportunity in AOSD patients in terms of clinical and laboratory disease control along with corticosteroid-sparing effect.

**Results:**

Nine AOSD patients (8 females and 1 male) treated with CAN for 15.00 ± 12.3 months were enrolled. Resolution of clinical manifestations was reported in 8/9 cases at the 3-month assessment; a significant decrease in the number of tender joints (*p* = 0.009), swollen joints (*p* = 0.027), and disease activity score on 28 joints-C-reactive protein (DAS28-CRP) (*p* = 0.044) was observed during the study period. The systemic score of disease activity significantly decreased at the 3-month and 6-month assessments and at the last visit compared to the start of treatment (*p* = 0.028, *p* = 0.028, and *p* = 0.018, respectively). The daily corticosteroid dosage was significantly reduced at the 3-month and at the last follow-up visits (*p* = 0.017 and *p* = 0.018, respectively). None of the patients experienced adverse events or severe adverse events during the follow-up.

**Conclusions:**

CAN has shown prompt and remarkable effectiveness in controlling AOSD activity in a real-life contest, with a significant glucocorticoid-sparing effect and an excellent safety profile.

## 1. Introduction

Adult-onset Still's disease (AOSD) is a rare multisystemic disorder characterized by an only partially understood etiology. In addition to the involvement of adaptive immunity, recent studies have disclosed the pivotal role of the innate immune system and interleukin- (IL-) 1 in AOSD pathogenesis, thus inducing to include this clinical entity among polygenic multifactorial autoinflammatory diseases [1]. It is also considered as the “adult” counterpart of the systemic-onset juvenile idiopathic arthritis (SOJIA) [2, 3].

Clinical manifestations of AOSD are represented by spiking fever, often accompanied by an evanescent salmon-colored maculopapular rash, arthralgia and/or arthritis, myalgia, sore throat, splenomegaly and/or hepatomegaly, lymphadenomegaly, pleurisy, and/or pericarditis. Two different AOSD phenotypes have been described: a “systemic type,” characterized by predominantly systemic features and highly increased inflammatory markers, and a “chronic-articular type” including patients presenting with arthritis as a pivotal feature in addition to other AOSD manifestations and slightly increased inflammatory markers. The systemic type can be classified as either monocyclic or polycyclic, with the monocyclic form being characterized by a single inflammatory episode lasting from two months to one year and the polycyclic course featured by recurrent attacks and intercritic remission. Nonspecific laboratory abnormalities are found in AOSD, including an elevated erythrocyte sedimentation rate (ESR) and C-reactive protein (CRP), neutrophilic leukocytosis, mild to moderate increase in aminotransferase activity, and high serum ferritin levels [4].

Diagnosis is clinical and requires the exclusion of infectious, neoplastic, autoimmune, and other autoinflammatory diseases. Nowadays, many clinical criteria for AOSD classification are available, among which Yamaguchi et al.'s criteria are the most sensitive and Fautrel et al.'s criteria the most specific [5, 6]. On the other hand, the evaluation of disease severity is currently based on the application of a systemic score proposed by Pouchot and colleagues and later modified by Rau and colleagues. This score ranges from 0 to 12, according to the presence or absence of 12 AOSD-related manifestations, each scoring one point [7, 8].

First-line treatment is usually based on nonsteroidal anti-inflammatory drugs (NSAIDs), glucocorticoids, and corticosteroid-sparing drugs, mostly represented by conventional disease-modifying antirheumatic drugs (cDMARDs), as methotrexate and cyclosporine A. More recently, a beneficial role of IL-1 inhibitors in patients with AOSD or SOJIA has been described. The implementation of this class of biologic agents has shown remarkable effectiveness in managing clinical and laboratory AOSD manifestations, together with inducing a significant corticosteroid-sparing effect and improving patients' quality of life [9–12].

To date, experience with IL-1 blockers in AOSD relies on the use of the receptor antagonist anakinra (ANK) and the selective anti-IL-1*β* monoclonal antibody canakinumab (CAN). Nevertheless, real-life experience is mainly focused on the use of ANK, while data about CAN are quite limited and mainly based on case reports and small case series [9, 10, 12–15]. Therefore, we conducted the present retrospective study to evaluate the role of CAN as a therapeutic opportunity in patients affected by AOSD in routine clinical practice.

## 2. Patients and Methods

Patients diagnosed with AOSD and treated with CAN were consecutively enrolled in two Italian referring centers. Demographic, clinical, and therapeutic data were retrospectively collected from clinical records. The classification of AOSD was performed according to Yamaguchi's criteria applied at the time of diagnosis and referring to clinical and laboratory manifestations observed during attacks [5].

Before starting CAN, all patients had undergone a careful laboratory and radiologic screening in order to exclude other diseases mimicking AOSD, including infections, neoplasms, autoimmune diseases, and other autoinflammatory disorders. After the start of CAN, patients were closely monitored with quarterly clinical and laboratory assessments or in case of disease relapse or safety concerns.

The primary aim of the study was to assess the clinical and laboratory effect of CAN in controlling AOSD activity. The secondary aim of the study was to evaluate any decrease in the systemic score at the study time points along with the corticosteroid and cDMARD-sparing effect during follow-up. The ancillary aim was to describe the safety profile of CAN in AOSD patients.

The primary endpoint of the study corresponded to the control of both clinical (fever, skin rash, pleuritis, pericarditis, lymphadenopathy, liver involvement, pharyngodynia, and myalgia) and laboratory (CRP, ESR, ferritin serum level, and leukocytosis) AOSD-related manifestations at 3- and 6-month visits and at the last follow-up visit. Further primary endpoint corresponded to a statistically significant decrease in the number of tender joints, swollen joints, and the disease activity score on 28 joints- (DAS28-) CRP during the study period. Secondary endpoints were represented by a statistically significant decrease in the systemic score and in the daily corticosteroid dosage along with a decrease in the number of patients administered cDMARDs.

Daily corticosteroid posology was assessed as mg/day of prednisone or equivalent. Clinical, laboratory, and therapeutic data were assessed at the start of CAN treatment (baseline) and at the 3-month and 6-month assessments. The last assessment corresponded to the 9-month visit or the 12-month visit for 3 patients undergoing CAN for at least one year.

The study has been approved by the local Ethics Committee of Azienda Ospedaliera Universitaria Senese, Siena, Italy (Ref. N. 14951). The study protocol was conformed to the tenets of the Declaration of Helsinki; informed consent was obtained from all patients enrolled.

Descriptive statistics included sample sizes, mean, standard deviation (SD), median, and interquartile range (IQR) values. After having assessed normality distribution with the Shapiro-Wilk test, pairwise comparisons of qualitative data were performed using Student's *t*-test or Mann-Whitney *U* test or Wilcoxon test, as required. The SPSS software, version 24, was used for statistical computations, always considering a significance level of 95% (*p* value < 0.05); all tests performed were two-sided.

## 3. Results

Nine patients (8 females and 1 male) have been treated with CAN because of AOSD. All patients fulfilled Yamaguchi's classification criteria.  Table 1 summarizes demographic and clinical features recorded at the start of the treatment.

The median disease duration before starting CAN was 15 (IQR = 27) months. Patients had been previously administered with corticosteroids (*n* = 9, 100%), NSAIDs (*n* = 7, 77.8%), cDMARDs (*n* = 8, 88.9%), and other biologic agents (*n* = 5, 55.6%).  Table 2 provides details about previous and concomitant treatments at the start of CAN.

Five (55.6%) patients had previously been administered with ANK. In these cases, the first IL-1 inhibitor had been discontinued because of secondary inefficacy after 168 months of ANK treatment (*n* = 1), the occurrence of adverse events consisting in severe in situ urticarial skin reaction (*n* = 3), and long-term disease remission after 18 months of ANK treatment started at the standard dosage (100 mg/day) and progressively reduced to 100 mg every 7 days (*n* = 1). All these patients had experienced a new disease relapse before starting canakinumab.

Four (44.4%) patients were administered CAN as monotherapy; 9 (100%) patients started CAN at the dosage of 150 mg/4 weeks (1.8-2.2 mg/kg/4 weeks), but 2 (22.2%) patients required an increase of dosage to 300 mg/4 weeks (3.7 and 3.8 mg/kg/4 weeks) within 3 months from the start of treatment because of persistent although improved laboratory or clinical inflammatory manifestations. No decrease in the posology or frequency of administration was performed during the study period. The overall CAN treatment duration was 15.00 ± 12.3 months.

At the 3-month assessment, CAN had induced complete control of clinical and laboratory manifestations in 8 out of 9 (88.9%) cases. The case with persistent clinical AOSD activity at the 3-month assessment showed fever, myalgia, arthritis, and leukocytosis, which also persisted at the 6-month visit, leading to CAN discontinuation due to lack of efficacy. A second patient discontinued CAN after 45 months while on persistent disease remission.

**Figure 1:**
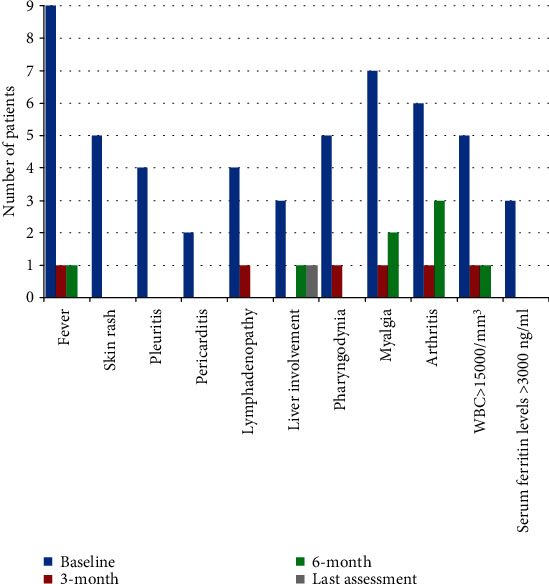
Frequency of adult-onset Still's disease clinical manifestations contributing to the systemic score proposed by Pouchot et al. [7] and modified by Rau et al. [8], referred to the start of canakinumab (baseline), the 3-month and 6-month visits, and the last assessment. Since one patient suspended canakinumab at the 6-month follow-up visit due to lack of efficacy, frequency counts refer to nine patients for baseline and 3-month and 6-month visits and to eight patients for the last assessment. Abbreviations: WBC: white blood cell.

**Figure 2:**
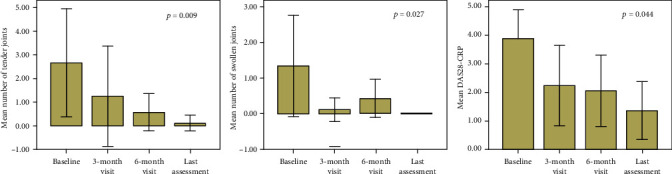
Trend of the mean number of tender joints, swollen joints, and disease activity score on 28 joints-C-reactive protein (DAS28-CRP) at the start of canakinumab (baseline) and at the three subsequent follow-up visits. Error bars refer to one standard deviation.

Figure 1 shows the frequency of resolution of specific clinical manifestations at different time points. Regarding joint involvement, a significant decrease in the number of tender joints (*p* = 0.009), swollen joints (*p* = 0.027), and DAS28-CRP (*p* = 0.044) was observed during the study period, as shown in Figure 2. In relation to the changes of DAS28-CRP, a significant decrease was observed at the 6-month visit (*p* = 0.028) and at the last assessment (*p* = 0.043) compared with the baseline, while no significant differences were observed between the baseline and the 3-month assessment (*p* = 0.09).

In 8 out of 9 patients, leukocytosis, ferritin serum levels, and inflammatory markers ESR and CRP turned to normal values within the 3-month assessment and to remain low in 6/9 patients at the 6-month visit and in 8/8 patients at the last assessment. The percentage of neutrophils at white cell count was higher than 80% in 8/9 patients at the start of treatment; conversely, none of the patients enrolled showed an increase compared to the reference ranges in the percentage of neutrophils at the 3-month, 6-month, and last assessment.

**Figure 3:**
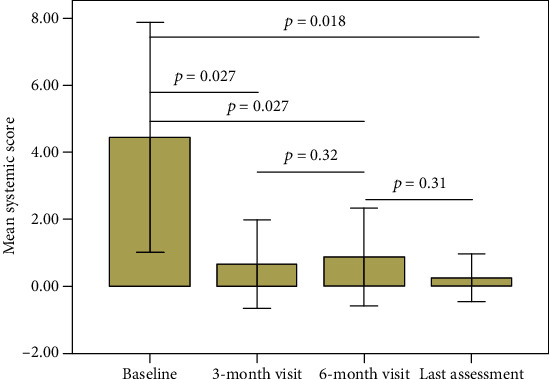
Changes in the systemic severity score proposed by Pouchot et al. and revised by Rau et al. at the different follow-up assessments. Horizontal lines and overlying *p* values describe pairwise comparisons between different time points, performed by using the Wilcoxon test. Error bars refer to one standard deviation.

The median systemic severity score was 4.0 (IQR = 7.0) at baseline, 0.0 (IQR = 1.00) at 3-month visit, 0.0 (IQR = 1.00) at 6-month visit, and 0.0 (IQR = 0.00) at the last visit. When compared to the start of CAN, the systemic severity score was significantly lower at the 3-month visit (*p* = 0.028), 6-month visit (*p* = 0.028), and last assessment (*p* = 0.018), with no significance between the 3- and the 6-month visits (*p* = 0.32).  Figure 3 describes the trend of the systemic score during the study period.

**Figure 4:**
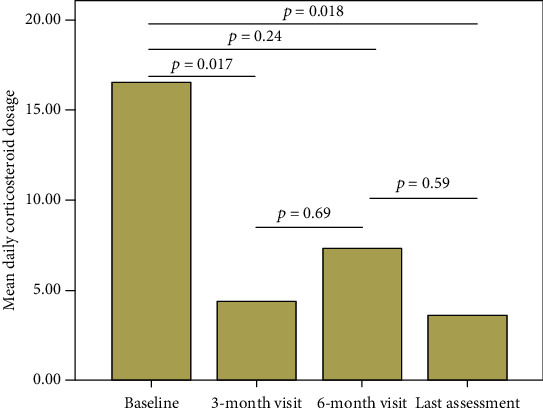
Mean daily corticosteroid dosage (prednisone or equivalent in mg/day) recorded at the start of canakinumab treatment and at the subsequent follow-up visits. Horizontal lines and overlying *p* values describe pairwise comparisons between different time points.

The median corticosteroid dosage was 15 (IQR = 25) mg/day at baseline, 5 (IQR = 5) mg/day at 3-month visit, 2.5 (IQR = 5) mg/day at 6-month visit, and 4 (IQR = 5) mg/day at the last assessment. The daily corticosteroid dosage was significantly reduced at the 3-month visit (*p* = 0.017) and at the last follow-up visit (*p* = 0.018), while no significant difference was observed between baseline and 6-month assessment (*p* = 0.24). Two (22.2%) patients suspended steroids within the 3-month assessment, and 3 (33.3%) patients stopped corticosteroids within the 6-month visit.  Figure 4 shows the mean systemic corticosteroid dosage at different time points.

Concomitant methotrexate was suspended in 1 out of 5 patients within 6 months from the start of CAN; a second patient interrupted methotrexate after 3 months from CAN introduction but later required the cDMARD reintroduction because of articular relapse. Conversely, none of the patients starting CAN as monotherapy underwent concomitant cDMARD introduction during follow-up. No differences were observed between patients starting CAN as monotherapy and patients concomitantly undergoing methotrexate in terms of disease duration before introducing CAN (*p* = 0.29) and the baseline systemic severity score (*p* = 0.41), while arthritis was significantly more frequent among patients requiring combination therapy compared to patients starting CAN as monotherapy (5/5 versus 1/4 patients, *p* = 0.048).

None of the patients experienced adverse events or severe adverse events during follow-up.

## 4. Discussion

When employed in AOSD patients, CAN has demonstrated to induce a full remission in about 70% of patients and at least a partial remission in about 90% of patients also in severe and resistant cases [3]. However, to the best of our knowledge, current literature is mainly based on case reports and small case series from nationwide surveys, reporting about the excellent role of CAN in inducing the control of AOSD flares, achieving disease remission, and permitting steroid-sparing effect. More recently, a pooled subgroup analysis on 29 old adolescents (aged >16 years), previously included in four clinical trials focused on SOJIA, confirmed the safety and the efficacy of CAN in such cases [16]. Based on these excellent results, in recent times, CAN has been approved as an on-label treatment approach for AOSD patients, thus recently widening its use in such a clinical framework. In this context, the present study represents the first attempt to assess the therapeutic role of CAN in AOSD patients based on real-life data and on a relatively wide number of cases.

The present study confirms the efficacy of CAN in controlling clinical and laboratory manifestations in the majority of AOSD patients, also inducing a significant and prompt decrease of the systemic severity score [7, 8]. Actually, all but one patient presented at the 3-month visit with the complete absence of AOSD-related manifestations. At the 6-month visit, two patients experienced a musculoskeletal flare, with arthritis and myalgia suddenly controlled by a temporary increase of corticosteroid dosage (from 5 to 20 mg/day in both cases); one patient showed a persistent increase in transaminase levels with no concomitant clinical or laboratory clues for AOSD activity. This suggested the occurrence of any other causes responsible for the increase of liver enzymes, including the concomitant use of methotrexate and/or fatty liver disease. Noteworthily, at the last follow-up assessment (after having excluded the patient with lack of efficacy), none of the patients presented with clinical or laboratory AOSD-related manifestations.

The excellent control of AOSD inflammatory features is also supported by the prompt and sustained decrease in the systemic severity score, which resulted significantly declined as soon as the 3-month follow-up visit.

Specifically, looking at the efficacy on articular items, a significant decrease in the number of tender joints, swollen joints, and DAS28-CRP values was identified during the study period. Noteworthily, the number of tender joints and the DAS28-CRP values progressively and steadily reduced over time. Indeed, only one out of nine patients showed swollen joints at the 3-month assessment, and none of the patients presented with arthritis at the last visit. On the other hand, articular exacerbation described in two patients at the 6-month visit proved to be temporary and easily manageable. As a whole, these data support a remarkable role of CAN in controlling the plethora of clinical manifestations, including articular involvement. This is in line with data reported by Cavalli et al., who described a dramatic clinical improvement on arthritis when CAN was used as a first-line treatment in AOSD patients [14]. Similarly, Feist et al. observed a significant reduction in active joint involvement from the baseline to the 85th day of CAN therapy in older adolescents and young adults affected by AOSD [16]. Of note, a very recent phase II randomized controlled trial has showed a not statistically significant difference in the frequency of patients reaching a DAS28 (ESR) reduction > 1.2 at week 12 between patients administered with CAN and those included in the placebo group. However, significantly higher American College of Rheumatology (ACR) 30%, ACR 50%, and ACR 70% response rates were observed among patients treated with CAN compared with those included in the placebo group [17]. This finding does not conflict with our own, since in our cohort of patients the improvement in articular manifestations proved to be persistent but progressive over time, with DAS28-CRP reduced in a statistically significant fashion starting from the 6-month assessment from the start of CAN.

Along with the optimal control of clinical manifestations, in the present study, laboratory assessment highlighted the prompt normalization of ferritin serum levels and inflammatory markers. In association with joint flares, which occurred in two patients after 6 months from the start of CAN, a slight increase in ESR and CRP values was recorded. Nevertheless, at the last assessment, all inflammatory markers lowered to range values in almost all patients. These findings are in line with previous experiences highlighting marked reductions in laboratory inflammatory markers and serum ferritin levels as a mirror of the efficacy on clinical manifestations [14, 16].

As for previous experiences, a significant corticosteroid-sparing effect was observed throughout the entire study period [15]. In detail, statistical significance was observed as soon as the 3-month visit, but not at the 6-month assessment due to the temporary increase in the daily corticosteroid dosage due to articular flares, as described above. Nevertheless, a statistically significant reduction in the corticosteroid dosage was observed again at the last assessment, confirming the remarkable steroid-sparing effect.

In our cohort, only one out of five patients could stop the concomitant use of methotrexate, while a second patient interrupting the cDMARD at the 3-month visit required the reintroduction of cDMARDs after articular relapse. Of note, only one patient administered with CAN monotherapy showed joint involvement, while all patients starting CAN plus methotrexate suffered from arthritis, thus suggesting a role of articular involvement in determining the need for combination therapy. Further studies on a wider number of patients should investigate this issue in the future.

Worth mentioning, five patients had been previously administered with ANK, with one patient requiring the switch to CAN after a loss of ANK efficacy, suggesting good clinical results for CAN also in patients losing efficacy to a first IL-1 inhibitor. A second patient started CAN owing to disease exacerbation despite a long-term disease remission induced by ANK. In this regard, future studies should clarify the best timing to interrupt IL-1 inhibitors in patients affected by polycyclic or chronic-articular AOSD, in order to avoid any potential disease reexacerbations.

Limitations of the study include the retrospective design and the small number of patients recruited. Furthermore, most of the patients underwent CAN at the posology of 150 mg every 4 weeks, corresponding to about 2 mg/kg/4 weeks. In this regard, we wonder whether the use of CAN at the posology of 4 mg/kg/4 weeks would have induced a faster and firmer control of disease manifestations, especially arthritis, and a higher sparing effect on concomitant cDMARDs.

In conclusion, CAN has shown prompt and remarkable effectiveness in controlling AOSD clinical and laboratory manifestations in a real-life contest, with a significant glucocorticoid-sparing effect and an excellent safety profile.

## Figures and Tables

**Table 1:** Clinical and laboratory features of patients enrolled at the start of canakinumab. Abbreviations: CAN: canakinumab; CRP: C-reactive protein; ESR: erythrocyte sedimentation rate; IQR: interquartile range; MAS: macrophage activation syndrome; SD: standard deviation.

Clinical features at the start of CAN	
Age at disease onset (years) (mean ± SD)	38.2 ± 12.8
Age at diagnosis (years) (mean ± SD)	38.7 ± 13.3
Systemic pattern, *n*	7 (77.8%)
Chronic-articular pattern	2 (22.2%)
Fever	9 (100%)
Skin rash	5 (55.6%)
Pleuritis	4 (44.4%)
Pneumonia	0 (0%)
Pericarditis	2 (22.2%)
Lymphadenopathy	4 (44.4%)
Liver involvement	3 (33.3%)
Pharyngodynia	5 (55.6%)
Myalgia	7 (77.8%)
Arthritis	6 (66.7%)
MAS	0 (0%)
Systemic Pouchot et al. score (mean ± SD)	4.4 ± 3.4
Laboratory investigations at the start of CAN	
Increased ferritin serum levels	5 (55.6%)
Increased leukocyte	5 (55.6%)
Increased ESR	6 (66.7%)
Increased CRP	7 (77.8%)
Increased transaminases	3 (33.3%)

**Table 2:** Treatments administered after disease onset and at the start of canakinumab. Abbreviations: CAN: canakinumab; cDMARDs: conventional disease-modifying antirheumatic drugs; *N*: number of patients.

	*N* (%)
Previous cDMARDs employed	
Methotrexate	8 (88.9)
Hydroxychloroquine	3 (33.3)
Cyclosporine A	2 (22.2)
Previous biologics employed	
Anakinra	5 (55.6)
Tocilizumab	3 (33.3)
Adalimumab	2 (22.2)
Etanercept	1 (11.1)
Treatments at the start of CAN	
Corticosteroids	9 (100)
cDMARDs (methotrexate)	5 (55.6)

## Data Availability

If required, data will be supplied by the corresponding author on request.
